# Skipping breakfast during pregnancy and hypertensive disorders of pregnancy in Japanese women: the Tohoku medical megabank project birth and three-generation cohort study

**DOI:** 10.1186/s12937-022-00822-9

**Published:** 2022-11-17

**Authors:** Misato Aizawa, Keiko Murakami, Ippei Takahashi, Tomomi Onuma, Aoi Noda, Fumihiko Ueno, Fumiko Matsuzaki, Mami Ishikuro, Taku Obara, Hirotaka Hamada, Noriyuki Iwama, Masatoshi Saito, Junichi Sugawara, Nobuo Yaegashi, Shinichi Kuriyama

**Affiliations:** 1 Graduate School of Medicine, Tohoku University, Sendai, Japan; 2 Tohoku Medical Megabank Organization, Tohoku University, Sendai, Japan; 3 Department of Pharmaceutical Sciences, Tohoku University Hospital, Sendai, Japan; 4 Department of Obstetrics and Gynecology, Tohoku University Hospital, Sendai, Japan; 5 International Research Institute of Disaster Science, Tohoku University, Sendai, Japan

**Keywords:** Skipping breakfast, Hypertensive disorders of pregnancy, Preeclampsia, Japan, Circadian clock

## Abstract

**Background:**

Hypertensive disorders of pregnancy (HDP) adversely affect the prognosis of mother and child, and the prognosis depends on the subtype of HDP. Skipping breakfast may be associated with increased blood pressure due to disruption of the circadian clock, but the association with the development of HDP has not been studied. The purpose of this study was to examine the association between skipping breakfast and the development of HDP and HDP subtypes in Japanese pregnant women.

**Methods:**

Of the pregnant women who participated in the Tohoku Medical Megabank Project Three-Generation Cohort Study, 18,839 who answered the required questions were included in the analysis. This study had a cross-sectional design. The breakfast intake frequency from pre-pregnancy to early pregnancy was classified into four groups: daily, 5–6 times per week, 3–4 times per week, and 0–2 times per week. HDP was classified into gestational hypertension (GH), chronic hypertension (CH), preeclampsia (PE), and severe preeclampsia (SuPE). Multiple logistic regression analysis and multinomial logistic analysis were used to calculate odds ratios (ORs) and 95% confidence intervals (CIs) for breakfast intake frequency and development of HDP or HDP subtypes. We performed a stratified analysis based on energy intake.

**Results:**

Of the participants, 74.3% consumed breakfast daily, and 11.1% developed HDP. Women who consumed breakfast 0–2 times per week had a higher risk of HDP (OR: 1.33, 95% CI: 1.14–1.56), CH (OR: 1.63, 95% CI: 1.21–2.19), and PE (OR: 1.68, 95% CI: 1.27–2.21) than those who consumed breakfast daily. No association was found between skipping breakfast and the risk of developing GH (OR: 1.26, 95% CI: 0.99–1.61) and SuPE (OR: 0.91, 95% CI: 0.55–1.49). Stratified analysis showed that the risk of developing HDP due to skipping breakfast was highest in the group with the highest daily energy intake.

**Conclusions:**

Skipping breakfast during pre-to early pregnancy is associated with the development of HDP. Further longitudinal studies are required to clarify the causal association between skipping breakfast and HDP.

**Supplementary Information:**

The online version contains supplementary material available at 10.1186/s12937-022-00822-9.

## Background

Hypertensive disorders of pregnancy (HDP) are the most frequent complications of pregnancy [1]. HDP accounts for 5–10% of all pregnancies, and maternal deaths due to HDP are estimated to be more than 70,000 per year worldwide [2].HDP is classified into four subtypes according to the development of hypertension, proteinuria, and organ failure syndrome: chronic hypertension (CH), gestational hypertension (GH), preeclampsia (PE), and superimposed preeclampsia (SuPE) [3]. In particular, PE is a major cause of maternal mortality [4] and is recognized as an independent risk factor for postpartum hypertension and cardiovascular disease (CVD) [1, 5]. PE has also been associated with diabetes mellitus [6] and dementia [7]. Therefore, HDP needs to be classified into subtypes, and its etiology and risk factors need to be clarified [8].

Although the etiology of HDP is not yet understood, there is some evidence to suggest that diet is a contributing factor [9, 10]. Dietary patterns, such as the Mediterranean diet, the Nordic diet, and the DASH diet, as well as vitamin D, calcium, and potassium intake, have been reported to be associated with a lower risk of developing HDP and PE [11–16]. The timing of dietary intake may also be associated with the development of HDP. Humans have a circadian clock that maintains basic life activities, and during pregnancy, the circadian clock changes due to hormonal and other physiological changes [17]. Since the circadian clock is affected by light, feeding, and exercise, it is important to regularly consume food to regulate the circadian rhythm [18, 19]. In addition, blood pressure follows the circadian clock, and disruption of the circadian clock is associated with hypertension [20, 21]. However, in Japan, 20–30% of pregnant women miss breakfast [22, 23], and irregular dietary intake, such as skipping breakfast, is associated with an increased risk of gestational diabetes and preterm birth [22, 24]. However, to the best of our knowledge, no studies have examined the association between skipping breakfast and the development of HDP or HDP subtypes in pregnant women.

Considering the above circumstances, we hypothesized that circadian rhythm disruption due to skipping breakfast in pregnant women is associated with the development of HDP. This study aimed to investigate the association between skipping breakfast and the development of HDP or HDP subtypes among pregnant Japanese women.

## Methods

**Fig. 1:**
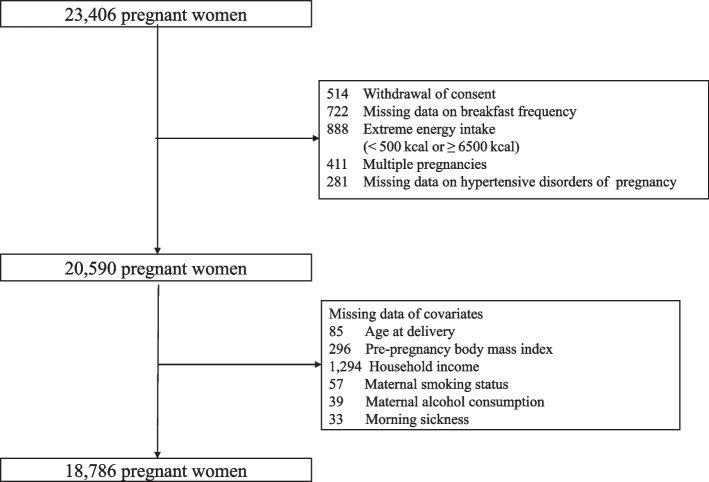
Flowchart of participant inclusion

We used data from the Tohoku Medical Megabank Project Birth and Three-Generation Cohort Study (TMM BirThree Cohort Study), the details of which have been provided elsewhere [25, 26]. This study had a cross-sectional design. From 2013 to 2017, pregnant women and their families were recruited at obstetrician-gynecologist clinics or hospitals where deliveries were scheduled. About 50 obstetric clinics and hospitals in Miyagi Prefecture participated in the recruitment. A total of 23,406 pregnant women were enrolled, of whom 514 withdrew consent, 722 had missing data on breakfast intake frequency, 888 had extreme energy intake (< 500 kcal or ≥ 6500 kcal) [27], 411 had multiple pregnancies, and 281 had no medical records of HDP. Of the remaining 20,590 pregnant women, 1804 were excluded because they had missing values for age at delivery, pre-pregnancy BMI, household income, smoking status, alcohol consumption, and morning sickness. Finally, the remaining 18,786 pregnant women were included in the analysis (Fig. 1). The protocol of the TMM BirThree cohort study was reviewed and approved by the Ethics Committee of Tohoku University Tohoku Medical Megabank Organization (2013-1-103-1).

The breakfast intake frequency of pregnant women was obtained from the Food Frequency Questionnaire (FFQ), which was distributed during early pregnancy (0–13 weeks). The FFQ assessed the frequency and quantity of 130 foods and beverages consumed in the past year. Energy-adjusted nutrient intake was calculated using a residual method [28]. The breakfast intake frequency was assessed using the question, “How often do you eat breakfast?”. There were six categories of intake frequency in the response items: less than once a month, 1–3 times a month, 1–2 times a week, 3–4 times a week, 5–6 times a week, and every day. The first three categories were combined into 0–2 times per week due to the small number of participants.

Blood pressure data were measured at maternity clinics and hospitals during prenatal check-ups. HDP was identified from maternal medical records, and HDP subtypes were identified using a proprietary algorithm that can extract HDP subtypes based on the American College of Obstetricians and Gynecologists [3]. The algorithm determined hypertension to be in women with systolic or diastolic blood pressure (BP) of 140/90 mmHg or higher at least once during pregnancy, and proteinuria of 1+ or higher at least once during pregnancy. The subtype of HDP was defined as follows: hypertension identified before 20 weeks of gestation was defined as chronic hypertension (CH), hypertension identified after 20 weeks gestation was defined as gestational hypertension (GH), gestational hypertension with proteinuria after 20 weeks of gestation was defined as preeclampsia (PE), and chronic hypertension with proteinuria was defined as superimposed preeclampsia (SuPE). The accuracy of the algorithm was assessed by physicians using the medical records [29].

Covariates were age at delivery, pre-pregnancy BMI, household income, smoking status, alcohol consumption, childbirth history, gestational diabetes, morning sickness, insomnia, and nutritional intake (energy, carbohydrates, potassium, sodium, vitamin D, and calcium). Age at delivery was categorized into four groups: < 25 years, 25–29 years, 30–34 years, and ≥ 35 years. BMI before pregnancy was classified into three groups: < 18.5 kg/m^2^, 18.5–24.9 kg/m^2^, and ≥ 25.0 kg/m^2^. Household income was categorized into three groups: less than 4 million yen, 4 million yen to 6 million yen, and more than 6 million yen. Smoking status was categorized into four groups: never smoked, quit before pregnancy, quit after pregnancy, and currently smoking. Drinking was categorized into three groups: never, former, and current. A history of gestational diabetes mellitus was confirmed from the medical records. Morning sickness was categorized into four groups: never, nausea only, able to eat with vomiting, and unable to eat with vomiting. Insomnia was defined as a score of ≥6 on the Japanese version of the Athens Insomnia Scale [30]. Nutrient intake was calculated using FFQ during early pregnancy.

Statistical analysis was performed using an analysis of variance for continuous variables and the chi-square test for categorical variables to examine the differences in characteristics of pregnant women according to their breakfast intake frequency. The association between maternal breakfast intake frequency and the risk of developing HDP was evaluated using multivariate logistic regression analysis, and the association with the risk of developing HDP subtypes was evaluated by multinomial logistic analysis. Odds ratios (ORs) and 95% confidence intervals (95% CIs) were calculated for each group compared with the group that ate breakfast daily. Three models were constructed to examine these associations. Model 1 was a crude model. Model 2 was adjusted for age at delivery, pre-pregnancy BMI, household income, smoking status, alcohol intake, parity, gestational diabetes, morning sickness, and sleep duration. Model 3 was adjusted for model 2 and nutrient intake (energy, carbohydrates, potassium, sodium, vitamin D, and calcium). In addition, to examine the possibility that the association between breakfast intake frequency and the development of HDP depends on energy intake, a stratified analysis by quartiles of energy intake was performed. All statistical analyses were performed using SAS version 9.4 (SAS Institute Inc., Cary, NC, USA). Statistical significance was set at *p* < 0.05.

## Results

Table 1 shows the characteristics of the mothers according to their breakfast intake frequency. The mean age at birth (standard deviation, SD) was 31.4 (4.9) years. Among the mothers, 74.2% ate breakfast daily, while 8.9% ate breakfast 0–2 times a week. Compared to mothers who ate breakfast daily, mothers who skipped breakfast more often were younger at birth, had lower household income, higher rates of smoking, first childbirth, and gestational diabetes, and slept less. The nutritional and food intake status of these mothers was low for all nutrients, including energy (Additional file 1: **Table 1_Supplementary Material**).

**Table 1:** Characteristics of participants

	Frequency of breakfast consumption								*p*-value^1^
	Everyday		5–6 times/week		3–4 times/week		0–2 times/week		
n (%) or mean (SD)									
Participants	13,945	(74.2)	1882	(10.0)	1284	(6.8)	1675	(8.9)	
Age at delivery									
<25 years	805	(5.8)	235	(12.5)	203	(15.8)	328	(19.6)	< 0.0001
25–29 years	3519	(25.2)	620	(32.9)	429	(33.4)	568	(33.9)	
30–34 years	5362	(38.5)	643	(34.2)	428	(33.3)	491	(29.3)	
≥35 years	4259	(30.5)	384	(20.4)	224	(17.5)	288	(17.2)	
Pre-pregnancy body mass index (kg/m^2^)									
<18.5	1772	(12.7)	249	(13.2)	175	(13.6)	248	(14.8)	< 0.0001
18.5–24.9	10,431	(74.8)	1376	(73.1)	904	(70.4)	1163	(69.4)	
≥25.0	1742	(12.5)	257	(13.7)	205	(16.0)	264	(15.8)	
Equivalent household income (/year)									
<4 million Japanese yen	4735	(34.0)	789	(41.9)	552	(43.0)	800	(47.8)	< 0.0001
4–5 million Japanese yen	4656	(33.4)	559	(29.7)	410	(31.9)	466	(27.8)	
≥6 million Japanese yen	4554	(32.7)	534	(28.4)	322	(25.1)	409	(24.4)	
Smoking status									
Never	8876	(63.7)	1053	(56.0)	644	(50.2)	727	(43.4)	< 0.0001
Quit before pregnancy	3347	(24.0)	440	(23.4)	296	(23.1)	332	(19.8)	
Quit after pregnancy	1538	(11.0)	320	(17.0)	278	(21.7)	494	(29.5)	
Current	184	(1.3)	69	(3.7)	66	(5.1)	122	(7.3)	
Alcohol consumption									
Never	6535	(46.9)	815	(43.3)	552	(43.0)	655	(39.1)	< 0.0001
Former	4692	(33.7)	691	(36.7)	453	(35.3)	668	(39.9)	
Current	2718	(19.5)	376	(20.0)	279	(21.7)	352	(21.0)	
Parity ≥1	8370	(60.0)	678	(36.0)	447	(34.8)	496	(29.6)	< 0.0001
Gestational diabetes	345	(2.5)	46	(2.4)	29	(2.3)	50	(3.0)	0.58
Morning sickness									
Never	1920	(13.8)	282	(15.0)	194	(15.1)	273	(16.3)	< 0.0001
Nausea only	6394	(45.9)	787	(41.8)	538	(41.9)	647	(38.6)	
Able to eat with vomiting	4210	(30.2)	615	(32.7)	398	(31.0)	527	(31.5)	
Unable to eat with vomiting	1421	(10.2)	198	(10.5)	154	(12.0)	228	(13.6)	
Insomnia	4601	(33.0)	718	(38.2)	511	(39.8)	715	(42.7)	< 0.0001

*SD* standard deviation

^1^Compared using the chi-square test for categorical variables and an analysis of variance for continuous variables

Table 2 shows the association between maternal breakfast intake frequency and HDP, with 11.1% of pregnant women developing HDP. Compared with mothers who consumed breakfast daily, mothers who consumed breakfast 5–6 times per week, 3–4 times per week, and 0–2 times per week had ORs (95% CI) for HDP of 1.49 (1.30–1.72), 1.30 (1.09–1.54), and 1.56 (1.35–1.81), respectively (Model 1) (*p* for trend < 0.001). After adjusting for age at delivery, pre-pregnancy BMI, household income, smoking status, alcohol consumption, parity, gestational diabetes, morning sickness, and insomnia, the ORs (95% CI) for HDP were 1.37 (1.19–1.59), 1.15 (0.96–1.38), and 1.38 (1.18–1.62) (Model 2) (*p* for trend < 0.001). Furthermore, after adjusting for consumption of energy, carbohydrates, potassium, sodium equivalent, and vitamin D, the ORs (95% CI) for HDP were 1.35 (1.17–1.57), 1.13 (0.94–1.35), and 1.35 (1.15–1.58) (model 3) (*p* for trend < 0.001).

**Table 2:** Association between maternal breakfast intake frequency and HDP

	Frequency of breakfast consumption							*p*-for trend^1^
	Everyday	5–6 times/week		3–4 times/week		0–2 times/week		
HDP								
n cases/n (%)	1397/13945 (10.0)	268/1882 (14.2)		162/1284 (12.6)		248/1675 (14.8)		
Model 1, OR(95% CI)	1.00	1.49	(1.30–1.72)	1.30	(1.09–1.54)	1.56	(1.35–1.81)	< 0.001
Model 2, OR(95% CI)	1.00	1.37	(1.19–1.59)	1.15	(0.96–1.38)	1.38	(1.18–1.62)	< 0.001
Model 3, OR(95% CI)	1.00	1.35	(1.17–1.57)	1.13	(0.94–1.35)	1.35	(1.15–1.58)	< 0.001

*HDP* hypertensive disorders of pregnancy, *OR* odds ratio, *CI* confidence interval

Model 1 was the crude model

Model 2 was adjusted for age at delivery, pre-pregnancy body mass index, household income, smoking status, alcohol consumption, parity, gestational diabetes, morning sickness, and insomnia

Model 3 was adjusted for the variables adjusted for in model 2 and consumption of energy, carbohydrate, potassium, sodium, vitamin D, and calcium

^1^*p*-for trends were calculated as trends across categories

Table 3 shows the association between maternal breakfast intake frequency and HDP subtypes. The percentages of GH, CH, PE, and SuPE were 4.6, 2.9, 3.0, and 1.5%, respectively. Compared with mothers who consumed breakfast daily, the ORs (95% CI) of GH for mothers who consumed breakfast 5–6 times a week, 3–4 times a week, and 0–2 times a week were 1.25 (0.99–1.56), 1.14 (0.87–1.50), and 1.27 (0.99–1.62), respectively (Model 3). The adjusted ORs (95% CI) for developing CH were 1.60 (1.22–2.09), 1.26 (0.89–1.78), and 1.64 (1.21–2.20) (Model 3). The adjusted ORs (95% CI) for PE were 1.34 (1.02–1.77), 0.95 (0.65–1.37), and 1.67 (1.27–2.20) (Model 3). The adjusted ORs (95% CI) for developing SuPE were 1.35 (0.91–1.99), 1.48 (0.94–2.28), and 0.91 (0.56–1.50) (Model 3).

**Table 3:** Association between maternal breakfast intake frequency and HDP subtypes

	Frequency of breakfast consumption							*p*-for ternd^1^
	Everyday	5–6 times/week		3–4 times/week		0–2 times/week		
GH								
n cases/n (%)	559/13107 (4.3)	98/1712 (5.7)		64/1188 (5.4)		92/1519 (6.1)		
Model 1, OR(95% CI)	1.00	1.36	(1.09–1.70)	1.28	(0.98–1.67)	1.45	(1.15–1.82)	< 0.001
Model 2, OR(95% CI)	1.00	1.26	(1.00–1.57)	1.15	(0.88–1.52)	1.29	(1.01–1.64)	0.03
Model 3, OR(95% CI)	1.00	1.25	(0.99–1.56)	1.14	(0.87–1.50)	1.27	(0.99–1.62)	0.04
CH								
n cases/n (%)	332/12880 (2.6)	72/1686 (4.3)		39/1161 (3.4)		61/1488 (4.1)		
Model 1, OR(95% CI)	1.00	1.69	(1.30–2.19)	1.31	(0.94–1.84)	1.62	(1.22–2.13)	< 0.001
Model 2, OR(95% CI)	1.00	1.65	(1.26–2.16)	1.29	(0.91–1.83)	1.65	(1.23–2.22)	< 0.001
Model 3, OR(95% CI)	1.00	1.60	(1.22–2.09)	1.26	(0.89–1.78)	1.64	(1.21–2.20)	0.003
PE								
n cases/n (%)	335/12883 (2.6)	66/1680 (3.9)		33/1155 (2.9)		75/1502 (5.0)		
Model 1, OR(95% CI)	1.00	1.53	(1.17–2.00)	1.10	(0.77–1.58)	1.97	(1.52–2.54)	< 0.001
Model 2, OR(95% CI)	1.00	1.35	(1.02–1.78)	0.95	(0.65–1.37)	1.66	(1.27–2.18)	0.003
Model 3, OR(95% CI)	1.00	1.34	(1.02–1.77)	0.95	(0.65–1.37)	1.67	(1.27–2.20)	0.004
SuPE								
n cases/n (%)	171/12719 (1.3)	32/1646 (1.9)		26/1148 (2.3)		20/1448 (1.4)		
Model 1, OR(95% CI)	1.00	1.46	(0.99–2.13)	1.63	(1.12–2.58)	1.03	(0.65–1.64)	0.13
Model 2, OR(95% CI)	1.00	1.38	(0.94–2.05)	1.51	(0.97–2.37)	0.95	(0.59–1.56)	0.37
Model 3, OR(95% CI)	1.00	1.35	(0.91–1.99)	1.48	(0.94–2.28)	0.91	(0.56–1.50)	0.54

*HDP* hypertensive disorders of pregnancy, *GH* gestational hypertension, *CH* chronic hypertension, *PE* preeclampsia, *SuPE* superimposed preeclampsia, *OR* odds ratio, *CI* confidence interval

Model 1 was the crude model

Model 2 was adjusted for age at delivery, pre-pregnancy body mass index, household income, smoking status, alcohol consumption, parity, gestational diabetes, morning sickness, and insomnia

Model 3 was adjusted for the variables adjusted for in model 2 and consumption of energy, carbohydrate, potassium, sodium, vitamin D, and calcium

^1^*p*-for trends were calculated as trends across categories

Table 4 shows the results of a stratified analysis conducted to determine whether the association between maternal breakfast frequency and HDP varied with energy intake. Energy intake was divided into quartiles, with the first quartile having the lowest nutrient intake and the fourth quartile having the highest nutrient intake (Additional file 1: Supplemental Table 1). In the first quartile of energy intake, the adjusted ORs (95% CI) of HDP for mothers who had breakfast 5–6 times a week, 3–4 times a week, and 0–2 times a week were 1.16 (0.87–1.55), 1.04 (0.75–1.44), and 1.10 (0.84–1.44). On the other hand, in the fourth quartile of energy intake, the adjusted ORs (95% CI) for HDP were 1.34 (0.99–1.80), 1.02 (0.69–1.50), and 1.66 (1.20–2.31).

**Table 4:** Association between maternal breakfast intake frequency and HDP stratified by energy intake

	Frequency of breakfast consumption							*p*-for trend^1^
	Everyday	5–6 times/week		3–4 times/week		0–2 times/week		
Energy intake								
Quartile 1 (low)								
n cases/n (%)	351/3091 (11.4)	72/509 (14.2)		53/394 (13.5)		95/702 (13.5)		
Model 1, OR(95% CI)	1.00	1.29	(0.98–1.69)	1.21	(0.89–1.65)	1.22	(0.96–1.56)	0.05
Model 2, OR(95% CI)	1.00	1.18	(0.88–1.57)	1.07	(0.77–1.47)	1.13	(0.87–1.47)	0.25
Model 3, OR(95% CI)	1.00	1.16	(0.87–1.55)	1.04	(0.75–1.44)	1.10	(0.84–1.44)	0.35
Quartile 2								
n cases/n (%)	327/3535 (9.2)	70/493 (14.2)		45/325 (13.9)		44/344 (12.8)		
Model 1, OR(95% CI)	1.00	1.62	(1.23–2.14)	1.58	(1.13–2.20)	1.44	(1.03–2.01)	< 0.001
Model 2, OR(95% CI)	1.00	1.53	(1.15–2.05)	1.44	(1.01–2.05)	1.21	(0.85–1.73)	0.03
Model 3, OR(95% CI)	1.00	1.48	(1.10–1.99)	1.39	(0.98–1.98)	1.17	(0.81–1.67)	0.05
Quartile 3								
n cases/n (%)	344/3675 (9.4)	62/432 (14.4)		30/282 (10.6)		48/308 (15.6)		
Model 1, OR(95% CI)	1.00	1.62	(1.21–2.17)	1.15	(0.78–1.71)	1.79	(1.29–2.48)	< 0.001
Model 2, OR(95% CI)	1.00	1.48	(1.10–2.01)	1.07	(0.71–1.61)	1.74	(1.22–2.48)	< 0.001
Model 3, OR(95% CI)	1.00	1.46	(1.07–1.98)	1.03	(0.68–1.56)	1.68	(1.17–2.40)	0.007
Quartile 4								
n cases/n (%)	375/3644 (10.3)	64/448 (14.3)		34/283 (12.0)		61/321 (19.0)		
Model 1, OR(95% CI)	1.00	1.45	(1.09–1.93)	1.19	(0.82–1.73)	2.05	(1.52–2.76)	< 0.001
Model 2, OR(95% CI)	1.00	1.35	(1.01–1.82)	1.04	(0.71–1.54)	1.72	(1.24–2.38)	0.003
Model 3, OR(95% CI)	1.00	1.34	(0.99–1.80)	1.02	(0.69–1.50)	1.66	(1.20–2.31)	0.005

*HDP* hypertensive disorders of pregnancy, *OR* odds ratio, *CI* confidence interval

Model 1 was the crude model

Model 2 was adjusted for age at delivery, pre-pregnancy BMI, household income, smoking status, alcohol consumption, parity, gestational diabetes, morning sickness, and insomnia

Model 3 was adjusted for the variables adjusted for in model 2 and consumption of energy, carbohydrate, potassium, sodium, vitamin D, and calcium

^1^*p*-for trends were calculated as trends across categories

## Discussion

This study showed that pregnant women who did not have breakfast daily during pre-to early pregnancy were at higher risk of developing HDP and HDP subtypes of CH and PE. Furthermore, in the group with the highest daily energy intake, pregnant women who skipped breakfast more frequently had the highest risk of developing HDP. To our knowledge, this is the first study to examine the association between skipping breakfast and the development of HDP in pregnant women.

Although no studies have reported on the association between breakfast intake and the risk of developing HDP, the association between breakfast intake and the risk of lifestyle-related diseases has been reported. In cohort studies, the risk ratio of low-frequency breakfast intake per week versus high frequency was 1.44 (95% CI 1.25–1.66) for overweight/obesity [31]. In a study of American adults, frequent breakfast intake was associated with a reduced risk of developing obesity, metabolic syndrome, hypertension, and type 2 diabetes during 18 years of follow-up compared to those with infrequent breakfast intake, but this result was not explained by the diet quality score [32]. As this evidence suggests, skipping breakfast may affect metabolic function.

There are three possible mechanisms by which skipping breakfast may affect the development of HDP. The first possible mechanism is the effect of breakfast intake on blood pressure elevation or suppression. A study of women aged 18–45 years in the US showed that skipping breakfast increased levels of the stress hormone cortisol, resulting in increased blood pressure and cardiovascular dysfunction [33]. A study of Australian adults showed that breakfast intake reduced systolic and diastolic blood pressure [34]. A study of Japanese adults showed that those who ate breakfast daily had lower blood pressure and a lower risk of stroke than those who did not consume breakfast daily [35]. Similar to these studies, the current study suggests that skipping breakfast may increase or not suppress blood pressure and affect the development of HDP.

The second possible mechanism is that the disruption of circadian rhythms due to skipping breakfast caused a decrease in metabolic function. The circadian clock is mainly controlled by two clocks: a central clock that regulates activity rhythms and a peripheral clock that regulates metabolic rhythms, and that eating in accordance with the body’s rhythms is important for maintaining normal circadian clock function [36, 37]. In a previous study of adults, the prevalence of hypertension was higher when energy intake after dinner was higher than breakfast [38], and that late-night meal intake disrupted circadian rhythms, reduced metabolic function, and increased blood pressure [39]. In the present study, the association between skipping breakfast and the development of HDP was strongest in the group with the highest daily energy intake. In the present study, skipping breakfast and eating high-energy meals in the afternoon and evening may be involved in the increased risk of HDP by causing a decrease in metabolic function due to disruption of circadian rhythms.

The third possible mechanism is that the disruption of circadian rhythms caused by skipping breakfast affects reproductive function. In previous studies, skipping breakfast in female college students was associated with dysmenorrhea [40–42], and pregnant women who experienced dysmenorrhea at a young age had a higher risk of developing HDP [43]. In a study using mice, it was observed that perinatal abnormalities such as placental abruption occurred when the Bmal1 clock gene, which regulates the circadian clock, was lost in utero [44]. These results suggest that disruption of the circadian clock by skipping breakfast may affect the hypothalamus, disrupt reproductive rhythms, and cause ovarian and uterine dysfunction [45]. In this study, the time of exposure to dysmenorrhea could not be identified; therefore, no association was found between dysmenorrhea and the development of HDP. However, skipping breakfast may have been associated with HDP development by affecting reproductive rhythms through the mechanisms reported in previous studies.

In the association between skipping breakfast and the development of HDP subtypes, there was an association between CH and PE, and no association between GH and SuPE. This suggests that skipping breakfast may be associated with hypertension both before and after pregnancy. Among them, PE is considered a two-stage disease theory in which placental dysplasia occurs in early pregnancy, causing placental ischemia and placental oxidative stress [46]; thus, skipping breakfast may also be involved in placental dysplasia. In contrast, in the present study, no association was found between skipping breakfast and the development of SuPE, the reason for which is unknown. The small number of cases compared to other disease types may have prevented us from obtaining a statistically significant association.

Regarding the association between frequency of breakfast consumption and the development of HDP, we identified an increased risk of HDP in those who consumed breakfast 5-6 times per week and those who consumed breakfast 0-2 times per week, whereas no association was found in those women who consumed breakfast 3-4 times per week. In a study of adult participants, BMI and incidence of metabolic syndrome increased with greater “social jet lag,” defined as the gap between weekday and weekend sleeping hours [47, 48]. Since “social jet lag” is associated with skipping breakfast, it is recommended that breakfast be consumed as regularly as possible every day [49]. Another study showed that those who consumed breakfast 0-2 times a week have an increased risk of stroke, while those who consumed breakfast 5-6 times a week show a U-shaped decrease in the risk of subarachnoid hemorrhage and coronary artery disease among Japanese adults [35]. The possible reason for the U-shaped association was that there may be other factors besides blood pressure that contribute to disease, even though breakfast intake reduces the risk of morning blood pressure elevation. The present study showed that consuming breakfast 5-6 times per week was associated with an increased risk of HDP. This finding suggests that daily breakfast intake may reduce “social jet lag”, and thereby reduce the risk of HDP. The present study also showed that the *P* value for the trend was < 0.001, despite the lack of association between those who consume breakfast 3-4 times per week and HDP after adjusting for covariates.

Regarding the dietary habits of breakfast skippers, skipping breakfast tends to be associated with unhealthy eating habits, such as having a high intake of processed foods [50]. The present study also found that those who consume breakfast 0-2 times per week have lower energy and nutrient intake, along with a higher intake of soft drinks, meat, and alcoholic beverages. Therefore, stratified analysis by energy intake showed that skipping breakfast and HDP risk increased in the group with higher energy intake. This suggests that the eating habits of skipping breakfast and having a robust lunch and dinner may increase HDP risk.

## Limitations

The present study has several limitations. First, this study was conducted in a part of Japan; thus, the findings cannot be generalized for other populations. The average age of the participants in the national survey of pregnant women in Japan was 31.0 years, whereas the average age of the participants in this study was 31.4 years, although the basic attributes were almost the same [51]. Second, the data on skipping breakfast were obtained from the self-reported questionnaires of the participants; thus, misinterpretation of skipping breakfast is inevitable. However, while the percentage of pregnant women skipping breakfast in Japan is 20–30% [22, 23], the percentage of women skipping breakfast was almost the same at 25.2% in the present study. Third, the types and amounts of food consumed for breakfast are unknown. To be able to better examine the association between skipping breakfast and the development of HDP, it would be useful to examine not only breakfast intake frequency but also the type, amount, and time of day of the meal. Fourth, since breakfast skipping is influenced by various social backgrounds, such as night work and time availability, the covariates adjusted for in this study may not fully exclude the effects of these backgrounds.

## Conclusion

In Japanese pregnant women, skipping breakfast before early pregnancy was associated with an increased risk of HDP and HDP subtypes CH and PE. This finding suggests that daily breakfast intake may reduce the risk of developing HDP. Aside from dietary intake of energy and nutrients, the timing of intake should also be considered to prevent HDP.

## Supplementary Information

**Additional file 1.**

## Data Availability

The TMM BirThree Cohort Study data that support the findings of this study are not publicly available due to them containing information that could compromise research participant consent. All inquiries about access to the data should be sent to the TMM (dist@megabank.tohoku.ac.jp).

## Abbreviations

BMI: Body mass index
CH: Chronic hypertension
FFQ: Food frequency questionnaire
GH: Gestational hypertension
HDP: Hypertensive disorders of pregnancy
JPHC study: Japan Public Health Center-Based study
95% CI: Confidence interval
OR: Odds ratio
PE: preeclampsia
SD: Standard deviation
SuPE: superimposed preeclampsia
TMM BirThree Cohort Study: Tohoku Medical Megabank Project Birth and Three-Generation Cohort Study
