# Supplementary material for: Skipping breakfast during pregnancy and hypertensive disorders of pregnancy in Japanese women: the Tohoku medical megabank project birth and three-generation cohort study
Source: Nutr J. 2022 Nov 17;21:71. doi: 10.1186/s12937-022-00822-9 (PMC9670553; doi:10.1186/s12937-022-00822-9)
Supplement: Supplementary file 1 — Additional file 1. [file 12937_2022_822_MOESM1_ESM.docx]

| **Online Supplementary Material**  Skipping breakfast during pregnancy and hypertensive disorders of pregnancy in Japanese women: The TMM BirThree Cohort Study.  First author: Misato Aizawa  **Supplementary Table 1.** Nutrient and dietary consumption by frequency of breakfast consumption | | | | | | | | |
| --- | --- | --- | --- | --- | --- | --- | --- | --- |
|  | Frequency of breakfast consumption | | | | | | | |
|  | Everyday | | 5-6 times/week | | 3-4 times/week | | 0-2 times/week | |
|  | n=13,945 | | n=1,882 | | n=1,284 | | n=1,675 | |
|  | mean (SD) | | | | | | | |
| Dietary and nutrient consumption^1^ |  |  |  |  |  |  |  |  |
| Energy consumption (kcal/day) | 1,704 | (578.1) | 1,645 | (579.8) | 1,631 | (632.5) | 1,528 | (660.6) |
| Protein consumption (g/day) | 58.0 | (10.7) | 55.9 | (10.7) | 55.5 | (12.1) | 54.6 | (12.9) |
| Lipid consumption (g/day) | 57.5 | (13.9) | 57.1 | (13.5) | 57.7 | (15.7) | 57.2 | (17.4) |
| Carbohydrate consumption (g/day) | 213.8 | (35.9) | 211.2 | (37.4) | 205.8 | (41.4) | 203.6 | (44.1) |
| Dietary fiber consumption (g/day) | 10.4 | (3.9) | 9.4 | (3.3) | 9.0 | (3.5) | 8.8 | (3.4) |
| Sodium consumption (mg/day) | 3,240 | (985.1) | 3,079 | (963.8) | 3,085 | (1011.5) | 3,012 | (1007.4) |
| Potassium consumption (mg/day) | 2,112 | (548.1) | 2,002 | (510.7) | 1,988 | (554.4) | 1,959 | (560.4) |
| Calcium consumption (mg/day) | 499.4 | (285.4) | 457.8 | (260.6) | 439.3 | (266.5) | 433.5 | (264.0) |
| Magnesium consumption (mg/day) | 217.2 | (45.8) | 205.9 | (42.9) | 203.1 | (43.8) | 201.1 | (47.1) |
| Phosphorus consumption (mg/day) | 932.1 | (216.6) | 888.3 | (198.0) | 877.5 | (223.0) | 862.4 | (222.4) |
| Iron consumption (mg/day) | 6.6 | (1.6) | 6.3 | (1.5) | 6.2 | (1.9) | 6.1 | (1.7) |
| Zinc consumption (mg/day) | 7.1 | (1.1) | 6.9 | (1.1) | 6.8 | (1.5) | 6.6 | (1.4) |
| Copper consumption (mg/day) | 1.0 | (0.2) | 0.9 | (0.2) | 0.9 | (0.2) | 0.9 | (0.2) |
| Vitamin D consumption (μg/day) | 5.5 | (4.6) | 5.1 | (4.1) | 5.0 | (3.8) | 4.8 | (3.6) |
| Vitamin K consumption (μg/day) | 228.6 | (139.9) | 199.0 | (105.3) | 191.4 | (102.1) | 184.7 | (100.9) |
| Vitamin B_1_ consumption (mg/day) | 0.8 | (0.2) | 0.8 | (0.2) | 0.8 | (0.2) | 0.8 | (0.2) |
| Vitamin B_2_ consumption (mg/day) | 1.1 | (0.4) | 1.0 | (0.4) | 1.0 | (0.5) | 1.0 | (0.4) |
| Vitamin B_6_ consumption (mg/day) | 1.1 | (0.2) | 1.0 | (0.2) | 1.1 | (0.3) | 1.0 | (0.3) |
| Vitamin B_12_ consumption (μg/day) | 4.5 | (2.8) | 4.4 | (4.4) | 4.5 | (3.3) | 4.4 | (2.6) |
| Folic acid consumption (μg/day) | 262.0 | (99.4) | 242.9 | (86.2) | 241.3 | (106.5) | 236.3 | (91.1) |
| Vitamin C consumption (mg/day) | 83.8 | (46.0) | 79.2 | (42.1) | 79.2 | (45.3) | 76.9 | (46.4) |
| Salt equivalent (g/day) | 8.2 | (2.5) | 7.8 | (2.5) | 7.8 | (2.6) | 7.6 | (2.6) |
| Cereal consumption (g/day) | 432.5 | (115.7) | 422.9 | (113.6) | 402.2 | (122.8) | 391.2 | (144.0) |
| Potato consumption (g/day) | 24.9 | (19.2) | 23.1 | (19.1) | 22.2 | (17.8) | 22.0 | (18.3) |
| Sugar consumption (g/day) | 0.8 | (2.3) | 1.1 | (3.2) | 1.4 | (3.3) | 1.4 | (3.1) |
| Bean consumption (g/day) | 60.4 | (62.5) | 56.4 | (56.8) | 51.5 | (58.6) | 56.0 | (60.7) |
| Seed consumption (g/day) | 0.7 | (2.8) | 0.7 | (1.7) | 0.8 | (2.0) | 0.8 | (2.1) |
| Vegetable consumption (g/day) | 158.3 | (116.8) | 141.3 | (92.8) | 139.9 | (92.7) | 134.3 | (88.2) |
| Fruit consumption (g/day) | 147.7 | (133.2) | 152.2 | (142.1) | 158.0 | (159.3) | 155.8 | (170.7) |
| Mushroom consumption (g/day) | 10.7 | (9.6) | 10.0 | (9.0) | 9.6 | (9.3) | 9.0 | (9.7) |
| Algae consumption (g/day) | 6.5 | (6.1) | 5.7 | (5.6) | 5.6 | (5.3) | 5.2 | (6.2) |
| Seafood consumption (g/day) | 37.9 | (33.8) | 36.0 | (37.5) | 35.9 | (31.0) | 34.4 | (30.8) |
| Meat consumption (g/day) | 75.0 | (45.8) | 79.4 | (43.6) | 86.3 | (52.5) | 86.1 | (53.5) |
| Egg consumption (g/day) | 29.0 | (29.2) | 26.9 | (26.6) | 26.5 | (29.2) | 25.3 | (24.2) |
| Milk products consumption (g/day) | 208.3 | (246.1) | 188.0 | (226.7) | 174.6 | (232.6) | 167.8 | (210.8) |
| Fat and oil consumption (g/day) | 10.3 | (5.1) | 10.0 | (4.5) | 10.0 | (4.5) | 9.6 | (4.4) |
| Confectionery consumption (g/day) | 18.5 | (19.5) | 20.1 | (20.2) | 19.6 | (18.5) | 21.5 | (25.2) |
| Alcoholic beverage consumption (g/day) | 95.2 | (239.6) | 126.7 | (258.2) | 162.6 | (335.1) | 196.9 | (421.1) |
| Soft drink consumption (g/day) | 40.6 | (89.1) | 57.4 | (116.0) | 69.1 | (131.4) | 84.7 | (162.2) |
| ^1^Energy-adjusted using the residual method except for maternal total energy consumption. | | | | | |  |  |  |
